# Temporal Trends in Heart Failure Incidence Among Medicare Beneficiaries Across Risk Factor Strata, 2011 to 2016

**DOI:** 10.1001/jamanetworkopen.2020.22190

**Published:** 2020-10-23

**Authors:** Rohan Khera, Nitin Kondamudi, Lin Zhong, Muthiah Vaduganathan, Joshua Parker, Sandeep R. Das, Justin L. Grodin, Ethan A. Halm, Jarett D. Berry, Ambarish Pandey

**Affiliations:** 1 Section of Cardiovascular Medicine, Department of Internal Medicine, Yale School of Medicine, New Haven, Connecticut; 2 Center for Outcomes Research and Evaluation, Yale New Haven Hospital, New Haven, Connecticut; 3 Division of Cardiology, Department of Internal Medicine, University of Texas Southwestern Medical Center, Dallas; 4 Department of Bioinformatics, University of Texas Southwestern Medical Center, Dallas; 5 Brigham and Women’s Hospital Heart and Vascular Center, Department of Medicine, Harvard Medical School, Boston, Massachusetts; 6 Department of Cardiovascular Medicine, Cleveland Clinic, Cleveland, Ohio; 7 Division of General Internal Medicine, Department of Internal Medicine, University of Texas Southwestern Medical Center, Dallas

## Abstract

**Importance:**

Heart failure (HF) incidence is declining among Medicare beneficiaries. However, the epidemiological mechanisms underlying this decline are not well understood.

**Objective:**

To evaluate trends in HF incidence across risk factor strata.

**Design, Setting, and Participants:**

Retrospective, national cohort study of 5% of all fee-for-service Medicare beneficiaries with no prior HF followed up from 2011 to 2016. The study examined annual trends in HF incidence among groups with and without primary HF risk factors (hypertension, diabetes, and obesity) and predisposing cardiovascular conditions (acute myocardial infarction [MI] and atrial fibrillation [AF]).

**Exposures:**

The presence of comorbid HF risk factors including hypertension, diabetes, obesity, acute MI, and AF identified by *International Classification of Diseases*, *Ninth Revision*, *Clinical Modification* codes and *International Statistical Classification of Diseases*, *Tenth Revision*, *Clinical Modification* codes.

**Main Outcomes and Measures:**

Incident HF, defined using at least 1 inpatient HF claim or at least 2 outpatient HF claims among those without a previous diagnosis of HF.

**Results:**

Of 1 799 027 unique Medicare beneficiaries at risk for HF (median age, 73 years [interquartile range, 68-79 years]; 56% female [805 060-796 253 participants during the study period]), 249 832 had a new diagnosis of HF. The prevalence of all 5 risk factors increased over time (0.8% mean increase in hypertension per year, 1.9% increase in diabetes, 2.9% increase in obesity, 0.2% increase in acute MI, and 0.4% increase in AF). Heart failure incidence declined from 35.7 cases per 1000 beneficiaries in 2011 to 26.5 cases per 1000 beneficiaries in 2016, consistent across subgroups based on sex and race/ethnicity. A greater decline in HF incidence was observed among patients with prevalent hypertension (relative excess decline, 12%), diabetes (relative excess decline, 3%), and obesity (relative excess decline, 16%) compared with those without corresponding risk factors. In contrast, there was a relative increase in HF incidence among individuals with acute MI (26% vs no acute MI) and AF (22% vs no AF).

**Conclusions and Relevance:**

Findings of this study suggest that the temporal decline in HF incidence among Medicare beneficiaries reflects a decrease in HF incidence among those with primary HF risk factors. The increase in HF incidence among those with acute MI and those with AF highlights potential targets for future HF prevention strategies.

## Key Points

**Question:**

What are the trends in heart failure (HF) incidence across strata of risk factor burden?

**Findings:**

In this national cohort study of 1 799 027 Medicare beneficiaries at risk for HF, the incidence of HF declined despite a concomitant increase in the prevalence of associated risk factors. This decline reflects a decrease in HF incidence among those with primary HF risk factors (hypertension, diabetes, and obesity).

**Meaning:**

Individuals with risk factors, including hypertension, diabetes, and obesity, had a temporal decline in HF incidence; however, the incidence of HF increased among those with prevalent predisposing cardiovascular conditions, highlighting a potential target population for further HF prevention.

## Teaser

This national cohort study evaluates trends in heart failure incidence across risk factor strata, including hypertension, diabetes, obesity, acute myocardial infarction, and atrial fibrillation among Medicare beneficiaries.

## Introduction

In the United States, Medicare beneficiaries represent 70% to 80% of all patients hospitalized with heart failure (HF) each year.^1^ The Medicare population has also experienced substantial changes in the epidemiology of HF, with progressively fewer Medicare beneficiaries being diagnosed as having HF each year over the last decade after several decades of increasing incidence.^2,3,4,5,6,7,8^ However, the epidemiological mechanisms underlying the observed decline in the incidence of HF are not well understood. This lack of understanding represents an important knowledge gap because the prevalence of HF among older individuals continues to increase despite the declining incidence, so the primary prevention of HF is a key strategy to reduce morbidity and mortality from HF in the United States.

A potential mechanism for the decline in the incidence of HF may be associated with a concomitant decrease in the prevalence of risk factors for HF. These include primary HF risk factors, such as hypertension, diabetes, and obesity, which may act through multiple distinct mechanisms to increase the risk of HF, and predisposing cardiovascular (CV) conditions, such as acute myocardial infarction (MI) and atrial fibrillation (AF), which can more directly lead to myocardial dysfunction and increase the risk of developing HF.^9^

Another mechanism underlying the reduction in the incidence of HF may be better treatment of individuals with prevalent risk factors,^10,11,12,13,14,15^ thereby mitigating the downstream development of HF. Better understanding of the epidemiological mechanisms contributing to the observed decline in the incidence of HF is important to develop future public health interventions aimed at preventing HF. Accordingly, in this study of a national cohort of Medicare beneficiaries, we evaluated the temporal trends in the incidence of HF across different strata of risk factor burden spanning inpatient and outpatient care settings.

## Methods

This retrospective, national cohort study was reviewed by the institutional review board of the University of Texas Southwestern Medical Center, which waived the requirement for informed consent because the study represents secondary analyses of deidentified data. The study followed the Strengthening the Reporting of Observational Studies in Epidemiology (STROBE) reporting guideline.

### Data Source

We used a national 5% sample of all fee-for-service Medicare beneficiaries with no prior HF followed up from 2011 to 2016, accessing data on all of the claims submitted to Medicare from inpatient and outpatient encounters, as well as physician or carrier claims. The data source has been described previously.^7^ Briefly, for every 100 individuals enrolled in Medicare, 5 are entered into the Medicare 5% sample based on a predefined combination of the Medicare beneficiary identification number, a 9-digit unique beneficiary identifier. Once entered into the Medicare 5% data set, the beneficiary is included in the data every year, with 5% of new Medicare beneficiaries entering the data set every year. Beneficiaries leave the sample only when they die or when they are no longer eligible for Medicare benefits. The data include information on all sampled Medicare beneficiaries but include inpatient and outpatient claims only for fee-for-service Medicare beneficiaries enrolled in Medicare Part A and Part B.

### Study Population

We created a cohort of Medicare beneficiaries 65 years and older who were continuously enrolled in both Part A and Part B of fee-for-service Medicare. Enrollment in Medicare Part A and Part B ensured that insurance claims submitted for all health care encounters for these patients were available. In this cohort, we classified patients as having prevalent HF if they had a health care encounter with a claim code for HF during the first 12 months of entering the cohort. The *International Classification of Diseases*, *Ninth Revision*, *Clinical Modification* codes for HF were 428.x, 402.x1, 404.x1, and 404.x3, and the *International Statistical Classification of Diseases*, *Tenth Revision*, *Clinical Modification* codes for HF were I50x, I11.0, I13, and I13.2. This approach assumes that patients with HF are likely to encounter the health care system either during a hospitalization or in an outpatient setting over the course of 1 year and has been used in previous studies^4,7^ using Medicare claims. Those without prevalent HF were followed up longitudinally within administrative claims to identify individuals who had new-onset or incident HF, which is defined in further detail below. We focused on the incidence of HF among this cohort in the contemporary era (2011-2016) after changes in data transaction standards for Medicare claims had been implemented widely.^16,17^ We used claims from 2009 and 2010 to identify prevalent HF for sensitivity analyses, as outlined in the Statistical Analysis section.

### Study Variables and Outcomes

The demographic characteristics of age, sex, and race/ethnicity were identified from the Medicare Denominator File. Sex and race/ethnicity categories are based on information provided by Medicare beneficiaries at the time of enrollment in Medicare. We used claims data over the 12-month period of entering the cohort to define the baseline characteristics and risk factor profile of individuals. We used inpatient, outpatient, and physician carrier claims to define 5 common risk factors that are identifiable in claims data. These include 3 conditions (hypertension, diabetes, and obesity) that serve as primary HF risk factors and potentially act through multiple pathways to elevate the risk of HF. An additional 2 conditions (acute MI and AF) were chosen as secondary predisposing CV conditions that may be associated with the presence of primary HF risk factors but also have a more proximate impact on the risk of developing HF. We used a single claim code for conditions across all sources to identify hypertension, diabetes, obesity, acute MI, and AF (eTable 1 in the Supplement). We also defined other comorbidities using a similar approach. These included all comorbidities that are listed in eTable 2 in the Supplement.

The primary study outcome was incident HF, which was defined as at least 1 hospitalization with an inpatient HF claim or at least 2 outpatient or carrier HF claims among individuals without prevalent HF. For the latter, we required the outpatient claims to be in 2 separate calendar quarters to limit the selection of individuals who had HF as a potential differential diagnosis rather than a diagnosis of HF that requires longitudinal care. This approach has been used in previous studies.^4,7^ In addition, among those with incident HF on follow-up, mortality within 30 days after the first diagnosis of HF was captured as the secondary study outcome.

### Statistical Analysis

The demographic and clinical characteristics of Medicare beneficiaries at risk of HF at the beginning of each calendar year between 2011 and 2016 are described as the median (interquartile range) for continuous variables and as percentages for categorical variables. The incidence rate of HF for each year was also calculated for the overall cohort and across subgroups based on sex and race/ethnicity. Annual trends in HF incidence rate were evaluated using linear regression models with year being the independent variable and yearly HF incidence being the dependent variable. In addition, trends in 30-day mortality rates of HF after the first diagnosis among those who developed incident HF during the study period between 2011 and 2016 were also calculated. To assess the association of comorbid conditions with the rate of incident HF, Poisson regression models were constructed with age, sex, race/ethnicity, socioeconomic status, and all 5 comorbid conditions as independent variables.

For each of the 5 comorbid conditions, HF incidence rate ratios between patients who had a comorbid condition and patients who did not have the condition were reported, adjusting for demographics and all other comorbid conditions. Separate models were created for each calendar year, and the annual trend of adjusted HF incidence rate ratios between patients with and without each of the comorbid conditions was also reported. To test the yearly trend, linear regression models were constructed with the HF incidence rate ratio in each year as the dependent variable and with calendar year as the independent variable. Finally, we evaluated risk-adjusted trends in mortality. For this analysis, we constructed a logistic regression model with 30-day mortality as the outcome, covariates included in the Centers for Medicare & Medicaid Services model for mortality as independent variables, and calendar year as the exposure, consistent with the approach used in previous studies.^18,19,20,21^

In sensitivity analyses, we defined prevalent HF based on a 2-year lookback period. Incidence trends were addressed only among those individuals who had 2 years of claims before a calendar year and no inpatient, outpatient, or professional claim for HF during this period.

The 2-sided level of statistical significance was set at *P* < .05 for all analyses. Statistical analysis was performed using R, version 3.6.0 (R Project for Statistical Computing).

## Results

**Figure 1.**
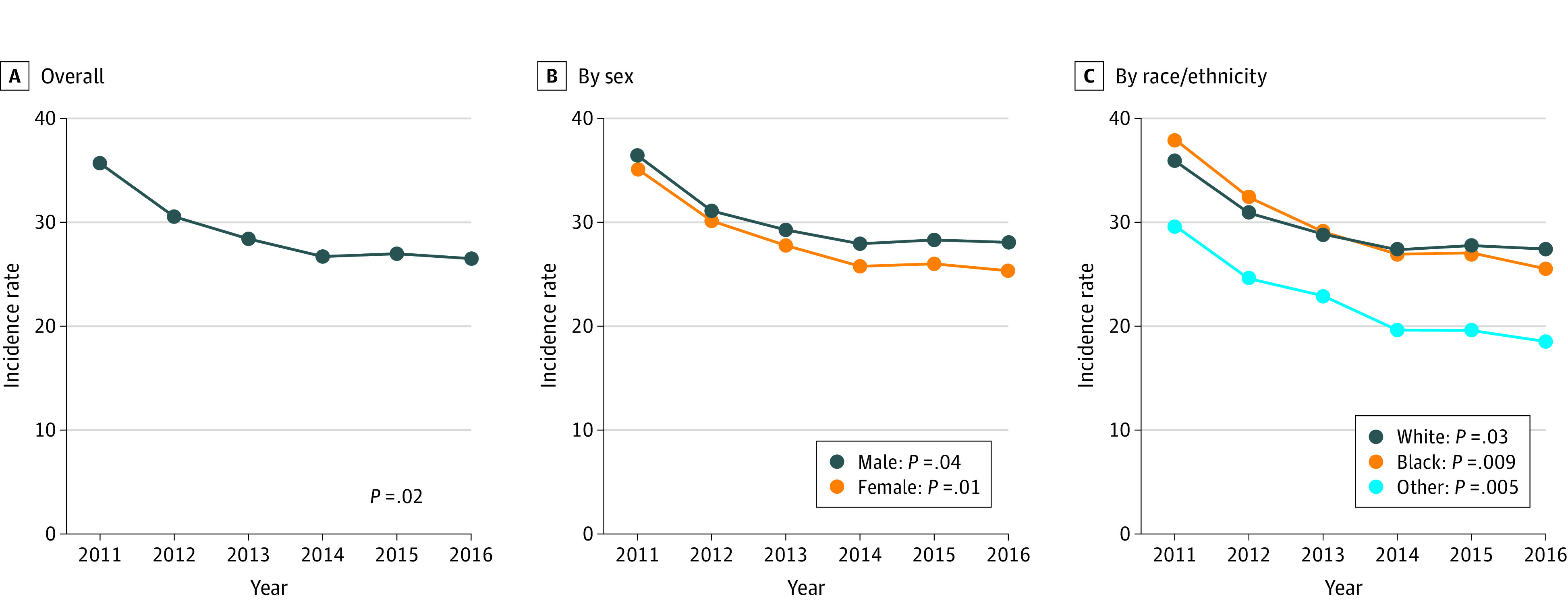
Incident Heart Failure Among Medicare Beneficiaries, 2011 to 2016 A-C, The incidence rate per 1000 Medicare beneficiaries is shown. *P* values reflect trends in the incidence rate per 1000 beneficiaries over time.

Overall, there were 1 799 027 unique Medicare beneficiaries at risk of developing incident HF at the beginning of each year during the study period. The median age of this cohort was 73 years (interquartile range, 68-79 years), 56% (805 060-796 253 participants during the study period) were female, 44% (621 196-620 718 participants during the study period) were male, and 18% had 1 or more risk factors for HF (eTable 2 in the Supplement). A total of 249 832 Medicare beneficiaries in our study cohort had a new diagnosis of HF during the 6-year study period. The prevalence of all 5 risk factors increased over time (0.8% mean increase in hypertension per year, 1.9% increase in diabetes, 2.9% increase in obesity, 0.2% increase in acute MI, and 0.4% increase in AF). The incidence of HF decreased over the study period from 35.7 cases per 1000 beneficiaries (n = 50 946) in 2011 to 26.5 cases per 1000 beneficiaries (n = 37 620) in 2016. This decline was observed across subgroups based on sex and race/ethnicity (Figure 1).

### Incidence of HF Across Risk Factor Strata

**Figure 2.**
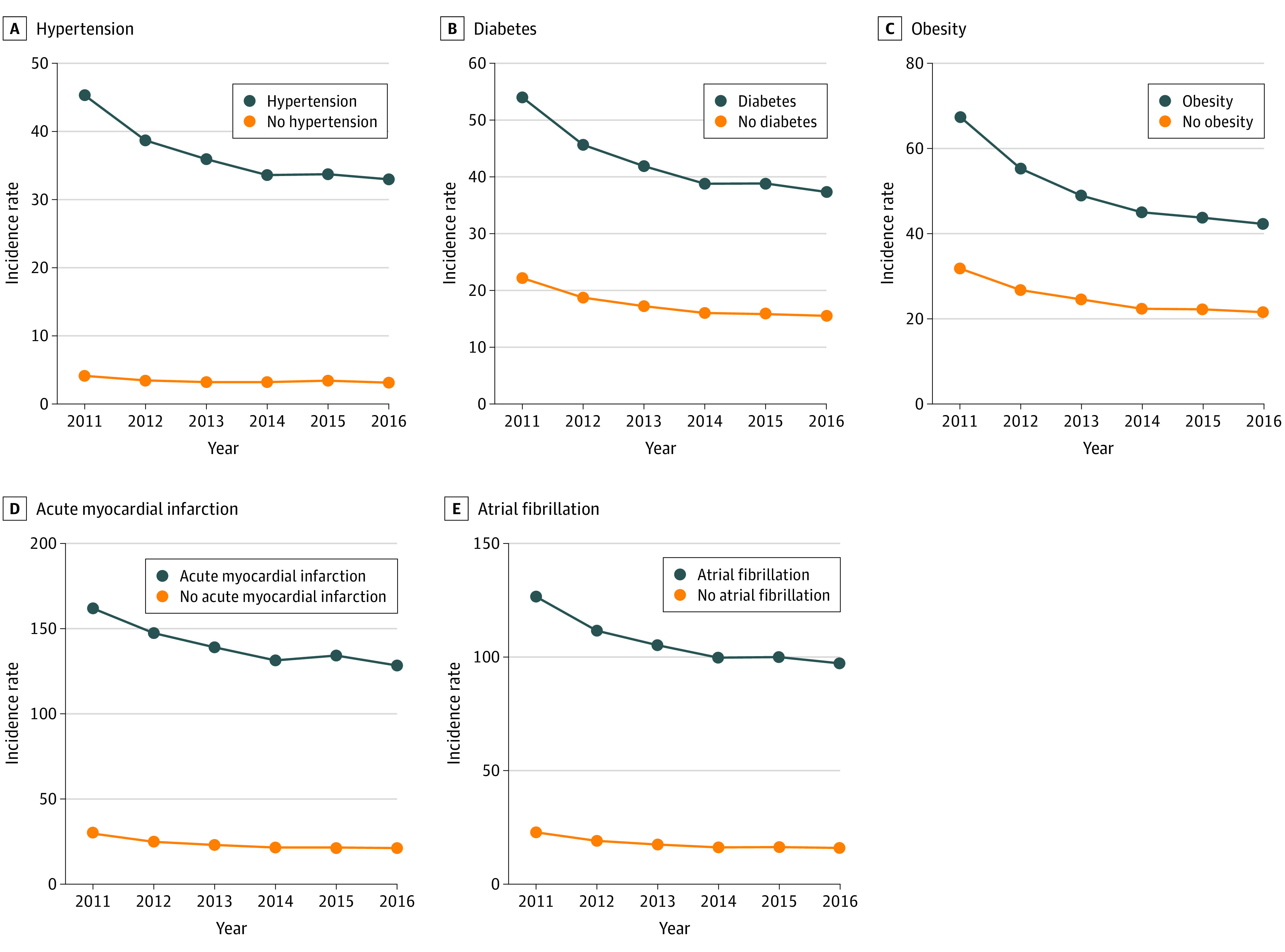
Incidence Rate of Heart Failure Among Medicare Beneficiaries by Comorbidity, 2011 to 2016 A-E, The incidence rate per 1000 Medicare beneficiaries is shown.

The prevalence of each risk factor (hypertension, diabetes, obesity, acute MI, and AF) increased over time among those with and without incident HF on follow-up (eFigure 1 in the Supplement). Throughout the study period, the incidence of HF was higher among individuals with vs without each of the risk factors for HF (Figure 2). Of all the risk factors, the incidence of HF was highest among individuals with acute MI and individuals with AF.

**Figure 3.**
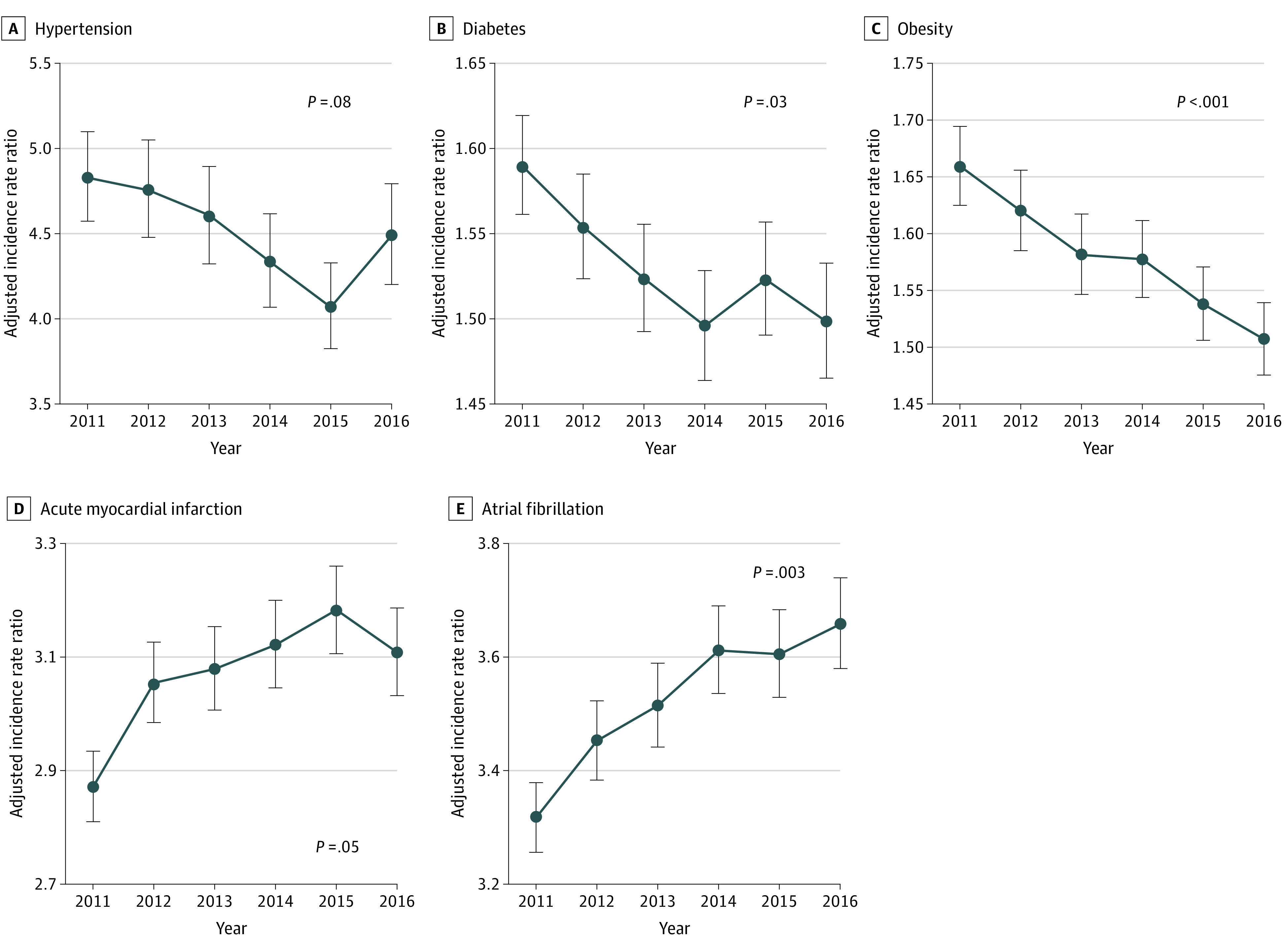
Adjusted Incidence Rate Ratios for Heart Failure Among Medicare Beneficiaries With vs Without Each Risk Factor Over Time, 2011 to 2016 A-E, Poisson regression models were constructed to assess the association of each risk factor with heart failure incidence for each year, with adjustment for age, sex, race/ethnicity, socioeconomic status, and all 5 risk factors. *P* values reflect trends in the incidence rate ratio over time. Error bars indicate 95% CIs.

During the study period, the incidence of HF declined across all risk factor–based subgroups, with a larger absolute decline among those with vs without each risk factor (Figure 2). For the primary HF risk factors, compared with the subgroup without a specific risk factor, there was a relative excess decline of 12% among patients with prevalent hypertension, 3% among patients with diabetes, and 16% among patients with obesity. In contrast, among individuals with predisposing CV conditions, there was a relative increase in HF incidence among individuals with acute MI (26% vs no acute MI) and individuals with AF (22% vs no AF) (Table). In adjusted analyses accounting for age, sex, race/ethnicity, socioeconomic status, and other risk factors, there was a statistically significant decrease in the incidence ratio among individuals with diabetes (adjusted incidence, 1.48; 95% CI, 1.45-1.51 to 1.37; 95% CI, 1.34-1.40) and individuals with obesity (adjusted incidence, 1.59; 95% CI, 1.55-1.62 to 1.42; 95% CI, 1.39-1.45) and a statistically significant increase among individuals with acute MI (adjusted incidence, 2.64; 95% CI, 2.58-2.70 to 2.81; 95% CI, 2.73-2.88) and individuals with AF (adjusted incidence, 3.02; 95% CI, 2.97-3.08 to 3.27; 95% CI, 3.20-3.35) during the study period compared with those without these risk factors (Figure 3).

**Table.** Change in Heart Failure Incidence Rates Among Medicare Beneficiaries Over the 6-Year Study Period in Patients With and Without Comorbidities^a^

Variable	Incidence of heart failure									
	Hypertension		Diabetes		Obesity		Acute myocardial infarction		Atrial fibrillation	
	No	Yes	No	Yes	No	Yes	No	Yes	No	Yes
2011	4.2	45.3	22.2	54.0	31.8	67.5	30.0	162.2	22.9	126.7
2016	3.2	33.0	15.6	37.3	21.6	42.3	21.6	128.5	16.1	97.3
% Relative change from 2011 to 2016	−24	−27	−30	−31	−32	−37	−28	−21	−30	−23
Relative excess change vs noncomorbid group, %	1 [Reference]	−12	1 [Reference]	−3	1 [Reference]	−16	1 [Reference]	26	1 [Reference]	22

^a^ Incidence rate per 1000 Medicare beneficiaries.

### Trends in HF Mortality Over Time

**Figure 4.**
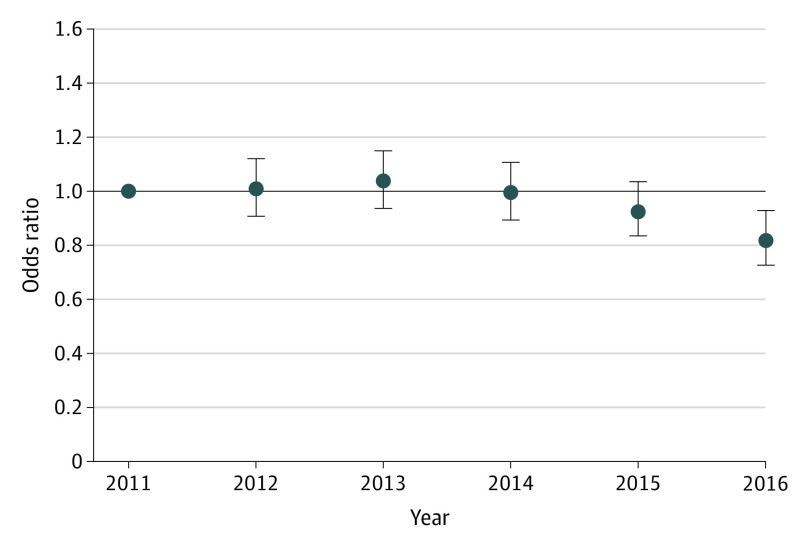
Risk-Adjusted Odds Ratio for 30-Day Mortality After a New Diagnosis of Heart Failure, With 2011 as the Reference Year Error bars indicate 95% CIs.

The unadjusted 30-day mortality rate after incident HF increased in the overall study cohort from 16.2 cases per 1000 Medicare beneficiaries to 17.2 cases per 1000 Medicare beneficiaries (eFigure 2 and eFigure 4 in the Supplement). In risk-adjusted analyses, the odds of 30-day mortality decreased over the study period (the risk-adjusted odds ratio for mean annual change relative to 2011 was 0.82; 95% CI, 0.73-0.93; *P* = .001) (Figure 4).

### Sensitivity Analyses

The use of a 2-year lookback period of claims to exclude prevalent HF was consistent with the primary analyses that used a 1-year period of claims. In the sensitivity analyses, we found a similar decline in incident HF, as well as similar trends among specific risk factor groups (eFigure 3 and eFigure 4 in the Supplement).

## Discussion

In a national cohort of almost 1.8 million fee-for-service Medicare beneficiaries 65 years and older followed up longitudinally for 6 years, we made several observations. The overall incidence of HF continued to decline between 2011 and 2016, regardless of sex or race/ethnicity. Concomitantly, among individuals with and without HF, rates of key underlying risk factors increased, suggesting that the decline in HF incidence is not because of an overall lower burden of HF risk factors. There was substantial heterogeneity in HF incidence across individual risk factors. Among patients with primary HF risk factors, including hypertension, diabetes, and obesity, the risk of developing new-onset HF compared with individuals without these risk factors decreased over time. In contrast, the risk of developing HF increased in individuals with predisposing CV conditions, such as acute MI and AF, compared with individuals without these conditions. In addition, we observed a concerning trend of increasing 30-day mortality rates among Medicare beneficiaries with HF throughout the study period. Taken together, these data suggest that the decline in HF incidence observed among Medicare beneficiaries is more likely associated with better treatment of patients with prevalent primary HF risk factors rather than early recognition of HF. Earlier identification and management of HF would be less consistent with the observed increase in 30-day mortality rates, a striking trend that warrants further investigation.

We found a statistically significant decline in incident HF during the study period. Our observation of a decreasing incidence of HF among Medicare beneficiaries was also noted in a prior study^7^ of these trends between 2004 and 2013. Previously, Curtis et al^4^ had reported a decreasing incidence of HF among Medicare beneficiaries between 1994 and 2003. Similar patterns were observed in the closely followed population of Olmsted County, Minnesota, where the incidence of HF declined between 2000 and 2010 by 37.5%.^22^ Such observations have also been found in international studies, notably from Scotland,^5^ Australia,^23^ Canada,^24^ Sweden,^25^ Denmark,^8^ Norway,^26^ and the United Kingdom,^27^ which all reported a decline in incident HF. eTable 3 in the Supplement summarizes these studies. Of note, the large study by Christiansen and colleagues^8^ reported a trend by age, with a decreasing incidence of HF among individuals older than 50 years but an increase among individuals 50 years and younger. Therefore, our findings among Medicare beneficiaries are consistent with these studies. The observed trends in incident and prevalent HF appear to be associated with the decline in HF mortality among individuals with incident HF over the last 2 decades.^28^

The declining incidence of HF among individuals with prevalent hypertension, diabetes, and obesity highlights the potential contribution of optimal management of these risk factors in the prevention of HF. Previous observational studies and randomized clinical trials have demonstrated that optimal control of these conditions is associated with lower risk of HF.^29,30,31^ Furthermore, data from Medicare Part D have shown an increase in the use of cardioprotective pharmacotherapies in patients with type 2 diabetes.^32^ Previous studies^33,34^ have also demonstrated an overall improvement in adherence to antihypertensive therapies among Medicare beneficiaries. It is plausible that such improvements in health care practices may have contributed to optimal management of these comorbidities, contributing to a decline in the incidence of HF.

In contrast to the decline in primary HF risk factors, we observed a statistically significant increase in incident HF in patients with established CV conditions of prior acute MI and AF. Several factors may underlie the observed increase in the incidence of HF over time among patients with prior MI. The lifetime risk of HF in patients after MI is high, such that 45% of individuals with prior MI develop HF by age 95 years.^35^ The survival of patients with MI has improved over time^36,37,38^ owing to better quality of care, including greater and more timely use of reperfusion interventions, more aggressive follow-up, and improved adherence to guideline-recommended therapies after MI.^39^ Furthermore, the use of diagnostic tests, such as echocardiograms, has increased among these patients.^40,41^ Consistent with our observations, previous studies^42,43^ have also demonstrated an increase in post-MI incidence of HF, with a concurrent decline in mortality after MI. Similarly, among patients with AF, improvement in life expectancy over time^44,45^ and the substantial overlap in symptom burden and pathophysiology between AF and HF^46,47^ may have contributed to the observed temporal increase in the incidence of HF among those with prevalent AF.

The relative increase in HF incidence among individuals with a history of acute MI or AF compared with those without these conditions highlights potential interventions aimed at prevention of HF. Management of patients with coronary artery disease and other risk factors, such as diabetes, with novel therapies like sodium-glucose cotransporter 2 inhibitors may reduce the downstream risk of HF.^48,49,50,51^ In addition, more effective and evidence-based management of AF with rhythm control strategies, such as catheter ablation, may also contribute to lower HF risk. Future studies are needed to assess whether greater uptake of these therapeutic strategies over time may alter the trajectory of HF incidence among these patient populations.

We also found that the unadjusted 30-day mortality rate after a new diagnosis of HF increased during the study period. However, this increase in mortality was explained by older age and increasing risk factor burden at diagnosis, with a small decrease in risk-adjusted mortality over this period. Of note, mortality trends in this study included only Medicare beneficiaries with incident HF, who represent a small proportion of the overall population of Medicare beneficiaries with HF. Therefore, these findings would not generalize to the larger trends in HF mortality among Medicare beneficiaries. However, the patterns found in our study of increasing risk factor burden have been observed in the larger Medicare population after hospitalization for HF.^20,21^ Furthermore, studies^18,19,52^ that have focused on mortality trends in more recent years have found a similar pattern of stable to decreasing mortality after HF hospitalization. Notably, studies^20,21,53^ that have investigated postdischarge mortality among Medicare beneficiaries with HF do not inform about the epidemiological trends in HF. Given the decrease in incident HF and the concurrent increase in HF prevalence, the life expectancy of patients with HF is expected to have increased. Although recent trends in life expectancy among patients with HF have not been explicitly evaluated, the overall hospitalization-related mortality (30-day postadmission mortality or combined in-hospital and postdischarge mortality) has decreased,^20,21,53^ a pattern consistent with an increasing prevalence and a decreasing incidence of HF.^28^

### Limitations

This study has some limitations. First, we used administrative claims to identify conditions of interest and patient outcomes and could not independently confirm these findings. However, as much as possible, we used claim codes that were used in prior studies to identify the major conditions included in the study, including HF, hypertension, diabetes, obesity, acute MI, and AF.^54,55^ Second, the study was sensitive to changes in coding intensity over time. Although there may have been pressure to code more conditions over time, the observed associations across conditions argue against this consideration being a major mechanism for the observed decline in HF incidence. Third, we could not definitively exclude prevalent HF among the study population. However, the results of the sensitivity analyses that evaluated a 2-year lookback period to define prevalent HF were consistent with the primary analyses. We argue that it is probably uncommon that an insured patient with HF would not seek any inpatient or outpatient care for comorbid HF. Fourth, we were unable to classify HF into subtypes because information on ejection fraction or left ventricular systolic dysfunction was not available. Fifth, although the study found a relative decrease in the incidence of HF among patients with hypertension, diabetes, and obesity, we cannot infer that this was associated with improvement in management, which was not evaluated in this study. This study only highlights that trends in HF incidence among major risk factor groups were concurrent with improvement in risk factor management during the last decade.^56^ Sixth, the study’s findings do not generalize to populations or individuals other than fee-for-service Medicare beneficiaries, and trends in younger populations, particularly those with high-risk conditions, were not a focus of this study.

## Conclusions

An observed decline in HF incidence among Medicare beneficiaries does not reflect a lower risk factor burden but rather a decrease in incident HF among those with associated risk factors, suggesting improved primary HF risk factor management. The relative increase in HF incidence among those with predisposing CV conditions, such as acute MI and AF, highlights potential targets for future HF prevention strategies.

## Notes

Supplement. **eTable 1.** International Classification of Diseases (ICD) 9 & 10 Codes Used to Identify Medicare Beneficiaries With Prevalent Primary and Secondary Heart Failure Risk Factors **eTable 2.** Clinical Characteristics of Medicare Beneficiaries Without Prevalent Heart Failure in the Beginning of Each Calendar Year During the Study Period **eTable 3.** Studies Reporting Heart Failure Incidence Trends **eFigure 1.** Percentage of Beneficiaries in Each Comorbid Group Among Those Who Developed Heart Failure and Those Who Did Not Develop Heart Failure, Yearly Trend **eFigure 2.** 30-Day Mortality Rate Among Beneficiaries With Incident Heart Failure Stratified by Sex and Race, Yearly Trend **eFigure 3.** Incident Rate of Heart Failure Among Beneficiaries by Comorbid Groups **eFigure 4.** Adjusted Incidence Rate Ratios for Heart Failure Among Beneficiaries With vs Without Each Risk Factor Over Time **eReferences.**

## References

[1] Chen J, Dharmarajan K, Wang Y, Krumholz HM National trends in heart failure hospital stay rates, 2001 to 2009. J Am Coll Cardiol. 2013;61(10):1078-1088. doi:10.1016/j.jacc.2012.11.057 23473413 PMC3939721

[2] Croft JB, Giles WH, Pollard RA, Casper ML, Anda RF, Livengood JR National trends in the initial hospitalization for heart failure. J Am Geriatr Soc. 1997;45(3):270-275. doi:10.1111/j.1532-5415.1997.tb00939.x 9063270

[3] Roger VL, Weston SA, Redfield MM, Trends in heart failure incidence and survival in a community-based population. JAMA. 2004;292(3):344-350. doi:10.1001/jama.292.3.344 15265849

[4] Curtis LH, Whellan DJ, Hammill BG, Incidence and prevalence of heart failure in elderly persons, 1994-2003. Arch Intern Med. 2008;168(4):418-424. doi:10.1001/archinternmed.2007.80 18299498

[5] Jhund PS, Macintyre K, Simpson CR, Long-term trends in first hospitalization for heart failure and subsequent survival between 1986 and 2003: a population study of 5.1 million people. Circulation. 2009;119(4):515-523. doi:10.1161/CIRCULATIONAHA.108.812172 19153268

[6] Roger VL Epidemiology of heart failure. Circ Res. 2013;113(6):646-659. doi:10.1161/CIRCRESAHA.113.300268 23989710 PMC3806290

[7] Khera R, Pandey A, Ayers CR, Contemporary epidemiology of heart failure in fee-for-service Medicare beneficiaries across healthcare settings. Circ Heart Fail. 2017;10(11):e004402. doi:10.1161/CIRCHEARTFAILURE.117.004402 29129828 PMC6057614

[8] Christiansen MN, Køber L, Weeke P, Age-specific trends in incidence, mortality, and comorbidities of heart failure in Denmark, 1995 to 2012. Circulation. 2017;135(13):1214-1223. doi:10.1161/CIRCULATIONAHA.116.025941 28174193

[9] Bui AL, Horwich TB, Fonarow GC Epidemiology and risk profile of heart failure. Nat Rev Cardiol. 2011;8(1):30-41. doi:10.1038/nrcardio.2010.165 21060326 PMC3033496

[10] Clemens KK, Shariff S, Liu K, Trends in antihyperglycemic medication prescriptions and hypoglycemia in older adults: 2002-2013. PLoS One. 2015;10(9):e0137596. doi:10.1371/journal.pone.0137596 26335938 PMC4559313

[11] Alexander GC, Sehgal NL, Moloney RM, Stafford RS National trends in treatment of type 2 diabetes mellitus, 1994-2007. Arch Intern Med. 2008;168(19):2088-2094. doi:10.1001/archinte.168.19.2088 18955637 PMC2868588

[12] Yoon SS, Gu Q, Nwankwo T, Wright JD, Hong Y, Burt V Trends in blood pressure among adults with hypertension: United States, 2003 to 2012. Hypertension. 2015;65(1):54-61. doi:10.1161/HYPERTENSIONAHA.114.04012 25399687 PMC11262548

[13] Gu Q, Burt VL, Dillon CF, Yoon S Trends in antihypertensive medication use and blood pressure control among United States adults with hypertension: the National Health and Nutrition Examination Survey, 2001 to 2010. Circulation. 2012;126(17):2105-2114. doi:10.1161/CIRCULATIONAHA.112.096156 23091084

[14] Minard LV, Corkum A, Sketris I, Fisher J, Zhang Y, Saleh A Trends in statin use in seniors 1999 to 2013: time series analysis. PLoS One. 2016;11(7):e0158608. doi:10.1371/journal.pone.0158608 27434392 PMC4951112

[15] Salami JA, Warraich H, Valero-Elizondo J, National trends in statin use and expenditures in the US adult population from 2002 to 2013: insights from the Medical Expenditure Panel Survey. JAMA Cardiol. 2017;2(1):56-65. doi:10.1001/jamacardio.2016.4700 27842171

[16] Ody C, Msall L, Dafny LS, Grabowski DC, Cutler DM Decreases in readmissions credited to Medicare’s program to reduce hospital readmissions have been overstated. Health Aff (Millwood). 2019;38(1):36-43. doi:10.1377/hlthaff.2018.05178 30615522

[17] Tsugawa Y, Figueroa JF, Papanicolas I, Orav EJ, Jha AK Assessment of strategies for managing expansion of diagnosis coding using risk-adjustment methods for Medicare data. JAMA Intern Med. 2019;179(9):1287-1290. doi:10.1001/jamainternmed.2019.1005 31242282 PMC6596333

[18] Wadhera RK, Joynt Maddox KE, Kazi DS, Shen C, Yeh RW Hospital revisits within 30 days after discharge for medical conditions targeted by the Hospital Readmissions Reduction Program in the United States: national retrospective analysis. BMJ. 2019;366:l4563. doi:10.1136/bmj.l4563 31405902 PMC6689820

[19] Khera R, Wang Y, Bernheim SM, Lin Z, Krumholz HM Post-discharge acute care and outcomes following readmission reduction initiatives: national retrospective cohort study of Medicare beneficiaries in the United States. BMJ. 2020;368:l6831. doi:10.1136/bmj.l6831 31941686 PMC7190056

[20] Wadhera RK, Joynt Maddox KE, Wasfy JH, Haneuse S, Shen C, Yeh RW Association of the Hospital Readmissions Reduction Program with mortality among Medicare beneficiaries hospitalized for heart failure, acute myocardial infarction, and pneumonia. JAMA. 2018;320(24):2542-2552. doi:10.1001/jama.2018.19232 30575880 PMC6583517

[21] Khera R, Dharmarajan K, Wang Y, Association of the Hospital Readmissions Reduction Program with mortality during and after hospitalization for acute myocardial infarction, heart failure, and pneumonia. JAMA Netw Open. 2018;1(5):e182777. doi:10.1001/jamanetworkopen.2018.2777 30646181 PMC6324473

[22] Gerber Y, Weston SA, Redfield MM, A contemporary appraisal of the heart failure epidemic in Olmsted County, Minnesota, 2000 to 2010. JAMA Intern Med. 2015;175(6):996-1004. doi:10.1001/jamainternmed.2015.0924 25895156 PMC4451405

[23] Teng TH, Finn J, Hobbs M, Hung J Heart failure: incidence, case fatality, and hospitalization rates in Western Australia between 1990 and 2005. Circ Heart Fail. 2010;3(2):236-243. doi:10.1161/CIRCHEARTFAILURE.109.879239 20071655

[24] Ezekowitz JA, Kaul P, Bakal JA, Quan H, McAlister FA Trends in heart failure care: has the incident diagnosis of heart failure shifted from the hospital to the emergency department and outpatient clinics? Eur J Heart Fail. 2011;13(2):142-147. doi:10.1093/eurjhf/hfq185 20959343

[25] Zarrinkoub R, Wettermark B, Wändell P, The epidemiology of heart failure, based on data for 2.1 million inhabitants in Sweden. Eur J Heart Fail. 2013;15(9):995-1002. doi:10.1093/eurjhf/hft064 23645498

[26] Sulo G, Igland J, Øverland S, Heart failure in Norway, 2000-2014: analysing incident, total and readmission rates using data from the Cardiovascular Disease in Norway (CVDNOR) Project. Eur J Heart Fail. 2020;22(2):241-248. doi:10.1002/ejhf.1609 31646725

[27] Conrad N, Judge A, Tran J, Temporal trends and patterns in heart failure incidence: a population-based study of 4 million individuals. Lancet. 2018;391(10120):572-580. doi:10.1016/S0140-6736(17)32520-5 29174292 PMC5814791

[28] Ziaeian B, Fonarow GC Epidemiology and aetiology of heart failure. Nat Rev Cardiol. 2016;13(6):368-378. doi:10.1038/nrcardio.2016.25 26935038 PMC4868779

[29] Folsom AR, Yamagishi K, Hozawa A, Chambless LE; Atherosclerosis Risk in Communities Study Investigators Absolute and attributable risks of heart failure incidence in relation to optimal risk factors. Circ Heart Fail. 2009;2(1):11-17. doi:10.1161/CIRCHEARTFAILURE.108.794933 19808310 PMC2637354

[30] Wright JT Jr, Williamson JD, Whelton PK, ; SPRINT Research Group A randomized trial of intensive versus standard blood-pressure control. N Engl J Med. 2015;373(22):2103-2116. doi:10.1056/NEJMoa1511939 26551272 PMC4689591

[31] Sundström J, Bruze G, Ottosson J, Marcus C, Näslund I, Neovius M Weight loss and heart failure: a nationwide study of gastric bypass surgery versus intensive lifestyle treatment. Circulation. 2017;135(17):1577-1585. doi:10.1161/CIRCULATIONAHA.116.025629 28258170 PMC5404408

[32] Sumarsono A, Everett BM, McGuire DK, Trends in aggregate use and associated expenditures of antihyperglycemic therapies among US Medicare beneficiaries between 2012 and 2017. JAMA Intern Med. 2019;180(1):141-144. doi:10.1001/jamainternmed.2019.3884 31566649 PMC6777260

[33] Tajeu GS, Kent ST, Kronish IM, Trends in antihypertensive medication discontinuation and low adherence among Medicare beneficiaries initiating treatment from 2007 to 2012. Hypertension. 2016;68(3):565-575. doi:10.1161/HYPERTENSIONAHA.116.07720 27432867 PMC5215087

[34] Sumarsono A, Everett BM, McGuire DK, Trends in aggregate use and associated expenditures of antihyperglycemic therapies among US Medicare beneficiaries between 2012 and 2017. JAMA Intern Med. 2019;180(1):141-144. doi:10.1001/jamainternmed.2019.3884 31566649 PMC6777260

[35] Pandey A, Omar W, Ayers C, Sex and race differences in lifetime risk of heart failure with preserved ejection fraction and heart failure with reduced ejection fraction. Circulation. 2018;137(17):1814-1823. doi:10.1161/CIRCULATIONAHA.117.031622 29352072 PMC6417883

[36] Johansson S, Rosengren A, Young K, Jennings E Mortality and morbidity trends after the first year in survivors of acute myocardial infarction: a systematic review. BMC Cardiovasc Disord. 2017;17(1):53. doi:10.1186/s12872-017-0482-9 28173750 PMC5297173

[37] Pandey A, Keshvani N, Khera R, Temporal trends in racial differences in 30-day readmission and mortality rates after acute myocardial infarction among Medicare beneficiaries. JAMA Cardiol. 2020:5(2):136-145. doi:10.1001/jamacardio.2019.4845 31913411 PMC6990949

[38] Yeh RW, Sidney S, Chandra M, Sorel M, Selby JV, Go AS Population trends in the incidence and outcomes of acute myocardial infarction. N Engl J Med. 2010;362(23):2155-2165. doi:10.1056/NEJMoa0908610 20558366

[39] Desai NR, Udell JA, Wang Y, Trends in performance and opportunities for improvement on a composite measure of acute myocardial infarction care. Circ Cardiovasc Qual Outcomes. 2019;12(3):e004983. doi:10.1161/CIRCOUTCOMES.118.004983 30871375

[40] Miller AL, Dib C, Li L, Left ventricular ejection fraction assessment among patients with acute myocardial infarction and its association with hospital quality of care and evidence-based therapy use. Circ Cardiovasc Qual Outcomes. 2012;5(5):662-671. doi:10.1161/CIRCOUTCOMES.112.965012 22949495

[41] Papolos A, Narula J, Bavishi C, Chaudhry FA, Sengupta PP U.S. hospital use of echocardiography: insights from the Nationwide Inpatient Sample. J Am Coll Cardiol. 2016;67(5):502-511. doi:10.1016/j.jacc.2015.10.090 26846948

[42] Ezekowitz JA, Kaul P, Bakal JA, Armstrong PW, Welsh RC, McAlister FA Declining in-hospital mortality and increasing heart failure incidence in elderly patients with first myocardial infarction. J Am Coll Cardiol. 2009;53(1):13-20. doi:10.1016/j.jacc.2008.08.067 19118718

[43] Velagaleti RS, Pencina MJ, Murabito JM, Long-term trends in the incidence of heart failure after myocardial infarction. Circulation. 2008;118(20):2057-2062. doi:10.1161/CIRCULATIONAHA.108.784215 18955667 PMC2729712

[44] Freeman JV, Wang Y, Akar J, Desai N, Krumholz H National trends in atrial fibrillation hospitalization, readmission, and mortality for Medicare beneficiaries, 1999-2013. Circulation. 2017;135(13):1227-1239. doi:10.1161/CIRCULATIONAHA.116.022388 28148599

[45] Schnabel RB, Yin X, Gona P, 50 Year trends in atrial fibrillation prevalence, incidence, risk factors, and mortality in the Framingham Heart Study: a cohort study. Lancet. 2015;386(9989):154-162. doi:10.1016/S0140-6736(14)61774-8 25960110 PMC4553037

[46] Santhanakrishnan R, Wang N, Larson MG, Atrial fibrillation begets heart failure and vice versa: temporal associations and differences in preserved versus reduced ejection fraction. Circulation. 2016;133(5):484-492. doi:10.1161/CIRCULATIONAHA.115.018614 26746177 PMC4738087

[47] Pandey A, Kim S, Moore C, ; ORBIT-AF Investigators and Patients Predictors and prognostic implications of incident heart failure in patients with prevalent atrial fibrillation. JACC Heart Fail. 2017;5(1):44-52. doi:10.1016/j.jchf.2016.09.016 28034376

[48] Zinman B, Wanner C, Lachin JM, ; EMPA-REG OUTCOME Investigators Empagliflozin, cardiovascular outcomes, and mortality in type 2 diabetes. N Engl J Med. 2015;373(22):2117-2128. doi:10.1056/NEJMoa1504720 26378978

[49] Neal B, Perkovic V, Mahaffey KW, ; CANVAS Program Collaborative Group Canagliflozin and cardiovascular and renal events in type 2 diabetes. N Engl J Med. 2017;377(7):644-657. doi:10.1056/NEJMoa1611925 28605608

[50] Wiviott SD, Raz I, Sabatine MS Dapagliflozin and cardiovascular outcomes in type 2 diabetes: reply. N Engl J Med. 2019;380(19):1881-1882. doi:10.1056/NEJMoa1812389 31067395

[51] Perkovic V, Jardine MJ, Neal B, ; CREDENCE Trial Investigators Canagliflozin and renal outcomes in type 2 diabetes and nephropathy. N Engl J Med. 2019;380(24):2295-2306. doi:10.1056/NEJMoa1811744 30990260

[52] Khera R, Wang Y, Nasir K, Lin Z, Krumholz HM Evaluation of 30-day hospital readmission and mortality rates using regression-discontinuity framework. J Am Coll Cardiol. 2019;74(2):219-234. doi:10.1016/j.jacc.2019.04.060 31296295 PMC8669780

[53] Khera R, Krumholz HM Effects of the Hospital Readmissions Reduction Program. Circ Cardiovasc Qual Outcomes. 2018;11(12):e005083. doi:10.1161/CIRCOUTCOMES.118.005083 30562071

[54] Krumholz HM, Normand SL, Wang Y Trends in hospitalizations and outcomes for acute cardiovascular disease and stroke, 1999-2011. Circulation. 2014;130(12):966-975. doi:10.1161/CIRCULATIONAHA.113.007787 25135276 PMC4171056

[55] Kucharska-Newton AM, Heiss G, Ni H, Identification of heart failure events in Medicare claims: the Atherosclerosis Risk in Communities (ARIC) study. J Card Fail. 2016;22(1):48-55. doi:10.1016/j.cardfail.2015.07.013 26211720 PMC4706484

[56] NCD Risk Factor Collaboration (NCD-RisC) Long-term and recent trends in hypertension awareness, treatment, and control in 12 high-income countries: an analysis of 123 nationally representative surveys. Lancet. 2019;394(10199):639-651. doi:10.1016/S0140-6736(19)31145-6 31327564 PMC6717084

