# Supplementary material for: Temporal Trends in Heart Failure Incidence Among Medicare Beneficiaries Across Risk Factor Strata, 2011 to 2016
Source: JAMA Netw Open. 2020 Oct 23;3(10):e2022190. doi: 10.1001/jamanetworkopen.2020.22190 (PMC7584929; doi:10.1001/jamanetworkopen.2020.22190)
Supplement: Supplement. — eTable 1. International Classification of Diseases (ICD) 9 & 10 Codes Used to Identify Medicare Beneficiaries With Prevalent Primary and Secondary Heart Failure Risk Factors eTable 2. Clinical Characteristics of Medicare Beneficiaries Without Prevalent Heart Failure in the Beginning of Each Calendar Year During the Study Period eTable 3. Studies Reporting Heart Failure Incidence Trends eFigure 1. Percentage of Beneficiaries in Each Comorbid Group Among Those Who Developed Heart Failure and Those Who Did Not Develop Heart Failure, Yearly Trend eFigure 2. 30-Day Mortality Rate Among Beneficiaries With Incident Heart Failure Stratified by Sex and Race, Yearly Trend eFigure 3. Incident Rate of Heart Failure Among Beneficiaries by Comorbid Groups eFigure 4. Adjusted Incidence Rate Ratios for Heart Failure Among Beneficiaries With vs Without Each Risk Factor Over Time eReferences. [file jamanetwopen-e2022190-s001.pdf]

Supplementary Online Content

Khera R, Kondamudi N, Zhong L, et al. Temporal trends in heart failure incidence among Medicare beneficiaries across risk factor strata, 2011 to 2016. *JAMA Netw Open*. 2020;3(10):e2022190. doi:10.1001/jamanetworkopen.2020.22190

eTable 1. International Classification of Diseases (ICD) 9 & 10 Codes Used to Identify Medicare Beneficiaries With Prevalent Primary and Secondary Heart Failure Risk Factors

eTable 2. Clinical Characteristics of Medicare Beneficiaries Without Prevalent Heart Failure in the Beginning of Each Calendar Year During the Study Period

eTable 3. Studies Reporting Heart Failure Incidence Trends

eFigure 1. Percentage of Beneficiaries in Each Comorbid Group Among Those Who Developed Heart Failure and Those Who Did Not Develop Heart Failure, Yearly Trend

eFigure 2. 30-Day Mortality Rate Among Beneficiaries With Incident Heart Failure Stratified by Sex and Race, Yearly Trend

eFigure 3. Incident Rate of Heart Failure Among Beneficiaries by Comorbid Groups

eFigure 4. Adjusted Incidence Rate Ratios for Heart Failure Among Beneficiaries With vs Without Each Risk Factor Over Time

eReferences.

This supplementary material has been provided by the authors to give readers additional information about their work.

© 2020 Khera R et al. *JAMA Network Open*.

eTable 1. International Classification of Disease (ICD) 9 & 10 Codes Used to Identify Medicare Beneficiaries with Prevalent Primary and Secondary Heart Failure Risk Factors

**Hypertension**	ICD 9 diagnoses including '4011','4019','4010','40200','40201','40210','40211','40290','40291','4030','40300','40301','4031','40310','40311','4039','40390','40391','4040','40400','40401','40402','40403','4041','40410','40411','40412','40413','4049','40490','40491','40492','40493','40501','40509','40511','40519','40591','40599','4372' or ICD 10 CCS diagnosis groups '98','99'
**Diabetes**	ICD9 diagnoses including '24900','25000','25001','7902','79021','79022','79029','7915','7916','V4585','V5391','V6546','24901','24910','24911','24920','24921','24930','24931','24940','24941','24950','24951','24960','24961','24970','24971','24980','24981','24990','24991','25002','25003','25010','25011','25012','25013','25020','25021','25022','25023','25030','25031','25032','25033','25040','25041','25042','25043','25050','25051','25052','25053','25060','25061','25062','25063','25070','25071','25072','25073','25080','25081','25082','25083','25090','25091','25092','25093') or ICD 10 CC diagnosis groups '49','50'
**Atrial Fibrillation**	ICD9 diagnoses including ‘42731' or ICD 10 diagnosis I48.xx
**Obesity**	ICD9 diagnoses including '2780','27800','27801','27802','27803','79391','V8521','V8522','V8523','V8524','V8525','V8530','V8531','V8532','V8533','V8534','V8535','V8536','V8537','V8538','V8539','V854','V8541','V8542','V8543','V8544','V8545','V8554','Z6841','Z6842','Z6843','Z6844','Z6845' or ICD 10 diagnosis group with E66
**Acute Myocardial Infarction**	ICD9 substrate CD1. 1,3) in ('410') and substrate CD1,5,5) not in ('2') or ICD 10 CCS diagnosis group ‘100’

Abbreviations: CCS, clinical classification software; ICD, International Classification of Disease

© 2020 Khera R et al. *JAMA Network Open*.

eTable 2. Clinical Characteristics of Medicare Beneficiaries Without Prevalent Heart Failure in the Beginning of Each Calendar Year During the Study Period[a]

	2011	2012	2013	2014	2015	2016
N	1,426,256	1,422,429	1,432,268	1,433,868	1,431,496	1,416,971
Age (y), median, IQR	73 (68,79)	73 (68,79)	73 (68,79)	73 (68,79)	73 (68,79)	73 (69,79)
Men, %	621196 (43.6)	621381 (43.7)	627489 (43.8)	629180 (43.9)	628102 (43.9)	620718 (43.8)
Race, N, %						
White	1219137 (85.5)	1213534 (85.3)	1218383 (85.1)	1215060 (84.7)	1208495 (84.4)	1192235 (84.1)
Black	118292 (8.3)	117147 (8.2)	117008 (8.2)	117074 (8.2)	116528 (8.1)	115047 (8.1)
Others	85265 (6)	86004 (6)	87590 (6.1)	88696 (6.2)	90039 (6.3)	90394 (6.4)
Unknown	3562 (0.2)	5744 (0.4)	9287 (0.6)	13038 (0.9)	16434 (1.1)	19295 (1.4)
Region, N, %						
Northeast	259523 (18.2)	256304 (18)	256241 (17.9)	254448 (17.7)	251785 (17.6)	247030 (17.4)
Midwest	338546 (23.7)	335989 (23.6)	336255 (23.5)	334731 (23.3)	332330 (23.2)	327981 (23.1)
South	549788 (38.5)	548933 (38.6)	553830 (38.7)	555981 (38.8)	556845 (38.9)	552658 (39)
West	257678 (18.1)	260442 (18.3)	265494 (18.5)	269115 (18.8)	272210 (19)	272638 (19.2)
Others	18875 (1.3)	18432 (1.3)	18066 (1.3)	17613 (1.2)	17080 (1.2)	16662 (1.2)
Comorbidity, N, %						
Myocardial Infarction	25987 (1.8)	32083 (2.3)	31353 (2.2)	30356 (2.1)	29725 (2.1)	29394 (2.1)
Peripheral Vascular Disease	109420 (7.7)	144026 (10.1)	139465 (9.8)	136529 (9.5)	134915 (9.4)	135626 (9.6)
Cerebrovascular Disease	119628 (8.4)	137364 (9.7)	133590 (9.3)	129848 (9.1)	125912 (8.8)	122278 (8.6)
Dementia	29968 (2.1)	40645 (2.9)	38113 (2.7)	36089 (2.5)	34600 (2.4)	47162 (3.3)
Chronic Pulmonary Disease	156977 (11)	187962 (13.2)	185654 (13)	184552 (12.9)	182267 (12.8)	184045 (13)
Rheumatic Disease	37874 (2.7)	43750 (3.1)	42816 (3)	41871 (2.9)	41445 (2.9)	41768 (3)
Peptic Ulcer Disease	11127 (0.8)	14703 (1)	14072 (1)	13404 (0.9)	12744 (0.9)	12341 (0.9)
Mild Liver Disease	31878 (2.2)	40875 (2.9)	41555 (2.9)	41572 (2.9)	42857 (3)	44438 (3.1)

© 2020 Khera R et al. *JAMA Network Open*.

Diabetes without Complication	274214 (19.2)	288508 (20.3)	285087 (19.9)	282276 (19.7)	277724 (19.4)	274126 (19.4)
Diabetes with Complication	54610 (3.8)	73515 (5.2)	74527 (5.2)	76440 (5.3)	78501 (5.5)	86855 (6.1)
Renal Disease	60669 (4.3)	82484 (5.8)	86578 (6.1)	91990 (6.4)	96516 (6.8)	102736 (7.3)
Cancer	136923 (9.6)	146272 (10.3)	144522 (10.1)	143560 (10)	142285 (10)	141619 (10)
AIDS/HIV	1055 (0.1)	1141 (0.1)	1197 (0.1)	1212 (0.1)	1270 (0.1)	1266 (0.1)
Charlson's score, median, IQR	0 (0,1)	0 (0,2)	0 (0,2)	0 (0,2)	0 (0,2)	0 (0,2)

© 2020 Khera R et al. *JAMA Network Open*.

eTable 3. Studies Reporting Heart Failure Incidence Trends (presented in chronologic order)

Author (Year)	Country	Study Period	Codes Used to Identify Heart Failure	Heart failure Incidence Trend
Curtis et al. (2008)[1]	United States	1994-2003	ICD-9: 428.XX, 402.X1, or 404.X3	32 per 1,000 to 29 per 1,000 persons (age-adjusted), from **1994 to 2003**
Jhund et al. (2009)[2]	Scotland	1986-2003	ICD-9 codes: 425.4, 425.5, 425.9, 428.0, 428.1, 428.9, or 402<br><br>ICD-10 codes: I50, I42.0, I42.6, I42.7, or I42.9)	Men: 1.2 per 1,000 to 1.1 per 1,000 persons<br>Women: 1.3 per 1,000 to 1 per 1,000 persons (age adjusted) from **1986 to 2003**
Teng et al (2010)[3]	Australia	1990-2005	ICD-9 codes: 428x, 402.01, 402.11, 402.91, 404.1, 404.3, 425x, 518.4, 514, 391.8, and 398.91<br><br>ICD-10 codes: I50x, I11.0, I13.0, I13.2, I42x, J81, I01.8, I020	Men: 1.9 per 1,000 to 1 per 1,000 persons<br>Women: 1.3 per 1,000 to 0.8 per 1,000 persons (age adjusted), from **1990 to 2005**
Ezekowitz et al. (2011)[4]	Canada	2000-2006	ICD-9 codes: 428.x<br>ICD-10 code: I50	5 per 1,000 to 4 per 1,000 persons (age-adjusted)<br>from **2000 to 2006**

© 2020 Khera R et al. *JAMA Network Open*.

Zarrinkoub et al (2013)[5]	Sweden	2006-2010	ICD-10 code: I50	Men: 4 per 1,000 to 3 per 1,000 persons Women: 4 per 1,000 to 3 per 1,000 persons (age adjusted) from **2006 to 2010**
Gerber et al (2015)[6]	United States	2000-2010	ICD-9 codes: 428, 425, 429.3, and 514	3 per 1,000 to 2 per 1,000 persons (age and sex adjusted) from **2000 to 2010**
Christiansen et al. (2017)[7]	Denmark	1995-2012	ICD-10 codes: DI50, DI110, DI42, DI43, DI130, DI132, DO903<br>ICD-8 codes: 427.09, 427.10, 427.11, 427.19, 428.99, 428.49	Adjusted (age and sex) incident rate ratios<br>≤50 years old: 1.02 (0.89–1.83) to 1.52 (1.33–1.73)<br>>50 years old: 1.02 (0.99–1.05) to 0.69 (0.67–0.71) from **1995 to 2012**
Khera et al. (2017)[8]	United States	2002-2013	ICD-9 codes: 428.x, 402.x1, 404.x1, and 404.x3	39 per 1,000 to 26 per 1,000 persons (age adjusted) **2002 to 2013**

© 2020 Khera R et al. *JAMA Network Open*.

Tsao et al (2018)[9]	United States	1990-2009	Incident heart failure diagnoses adjudicated by physician panel	20 per 1,000 to 19 per 1,000 persons from **1990–1999 to 2000–2009**
Conrad et al. (2018)[10]	United Kingdom	2002-2014	ICD-10 codes: I50.0, I50.1, I50.9, I42.0, I42.9, I11.0, I25.5, I13.2, I13.0<br>Primary care read codes: G581.00, G58.00, G580.11, G5yy900, G55..00, 585f.00, G58..11, G581.133, 33BA.00, 101..00, G5yyA00, G551.00, G41z.11, G58z.00, G554400, 662g.00, G581000, G580.12, G554300, 1O1..00, G580.14, G343.00, G58z.12, 662h.00, G582.00, G58z.00, G5yyD00, 6622f.00, G583.00, G580100, G555.00, G580.13, G55y.11, G580200, 8H2S.00, 8H2S.00, G554200, G583.12, G584.00, G554000, G580300, G559.00, G583.11, 8CMW800, G580400, G558100, Gyu5M00, G1yz100, 662i.00, 661M500, 8CMK.00, G21z100, G211100, G232.00, 8CeC.00	4 per 1,000 to 3 per 1,000 persons (age and sex adjusted), from **2002 to 2014**
Sulo et al. (2020)[11]	Norway	2000-2014	ICD-9 codes: 402.01, 402.11, 402.3, 402.7, 402.91, 425.4, 425.5, 425.9, 428, 428.x<br><br>ICD-10 codes: I09.81, I11.0, I50, and I50	Men: 2,055 to 1,805 persons<br>Women: 1,962 to 1,625 persons<br>Incident heart failure cases from **2000 to 2014**

© 2020 Khera R et al. *JAMA Network Open*.

eFigure 1. Percentage of Beneficiaries in Each Comorbid Group Among Those Who Developed Heart Failure and Those Who Did Not Develop Heart Failure, Yearly Trend. Hypertension (A), Diabetes (B), Obesity (C), Acute Myocardial Infarction (D), and Atrial Fibrillation (E). AF: Atrial fibrillation; AMI: Acute myocardial infarction.

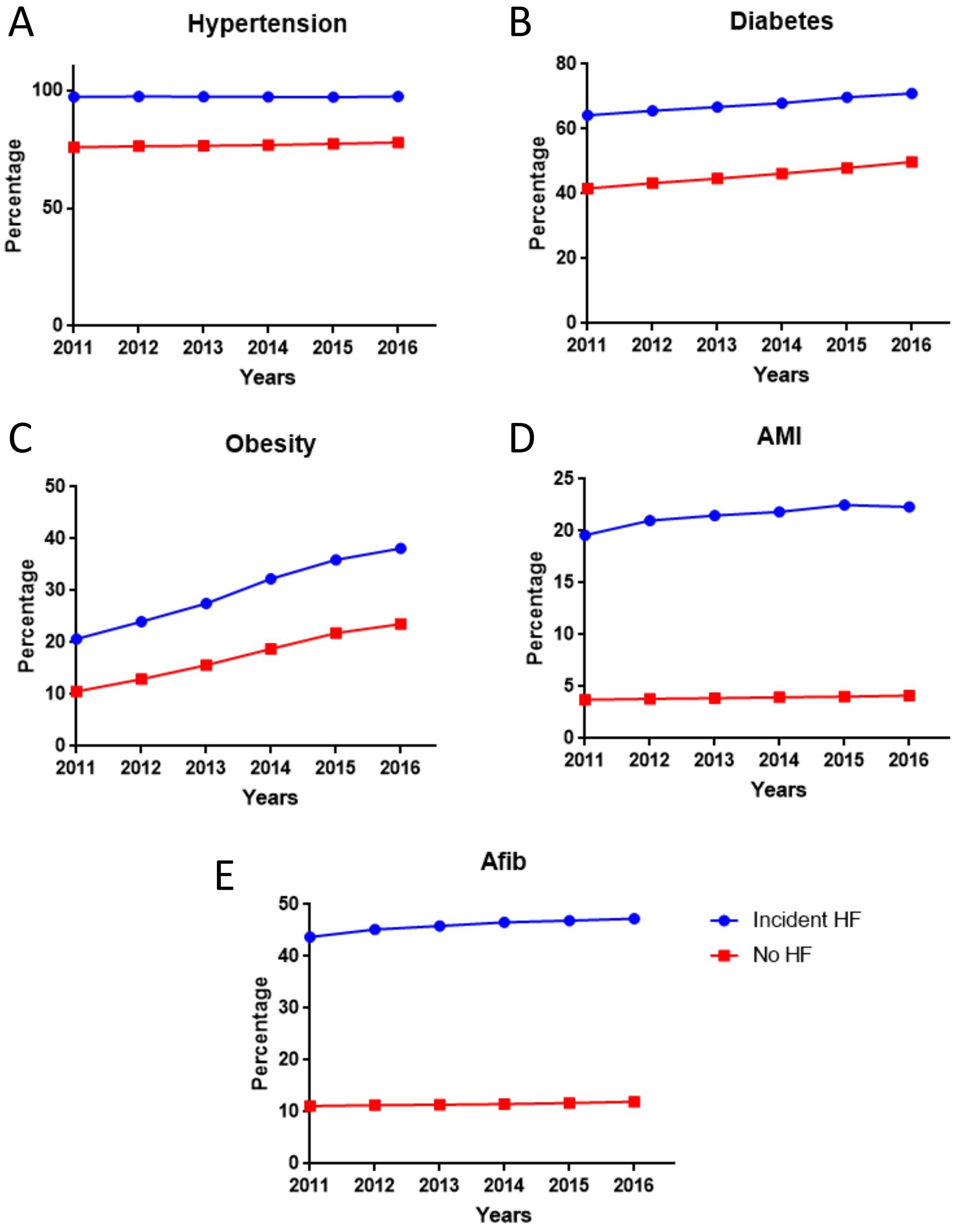

© 2020 Khera R et al. *JAMA Network Open*.

eFigure 2. 30-day Unadjusted Mortality Rate Among Beneficiaries with Incident Heart Failure Stratified by Sex (A) and Race (B), Yearly Trend. The p-value reflects trends in the mortality rate over time. (Mortality rate per 1000 persons)

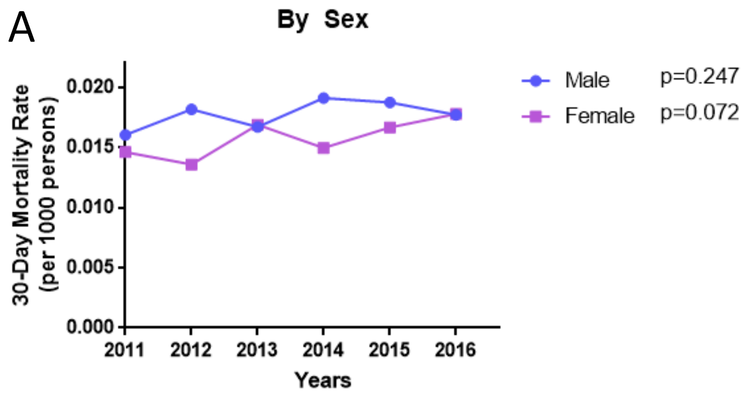

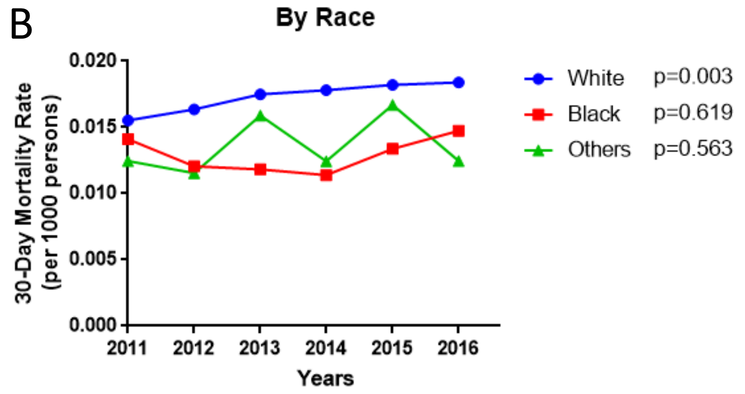

© 2020 Khera R et al. *JAMA Network Open*.

eFigure 3. Incident Rate of Heart Failure Among Beneficiaries by Comorbid Groups: Hypertension (A), Diabetes (B), Obesity (C), Acute Myocardial Infarction (D), and Atrial Fibrillation (E), Yearly Trend. AF: Atrial fibrillation; AMI: Acute myocardial infarction. 2-year period of claims used to exclude prevalent heart failure.

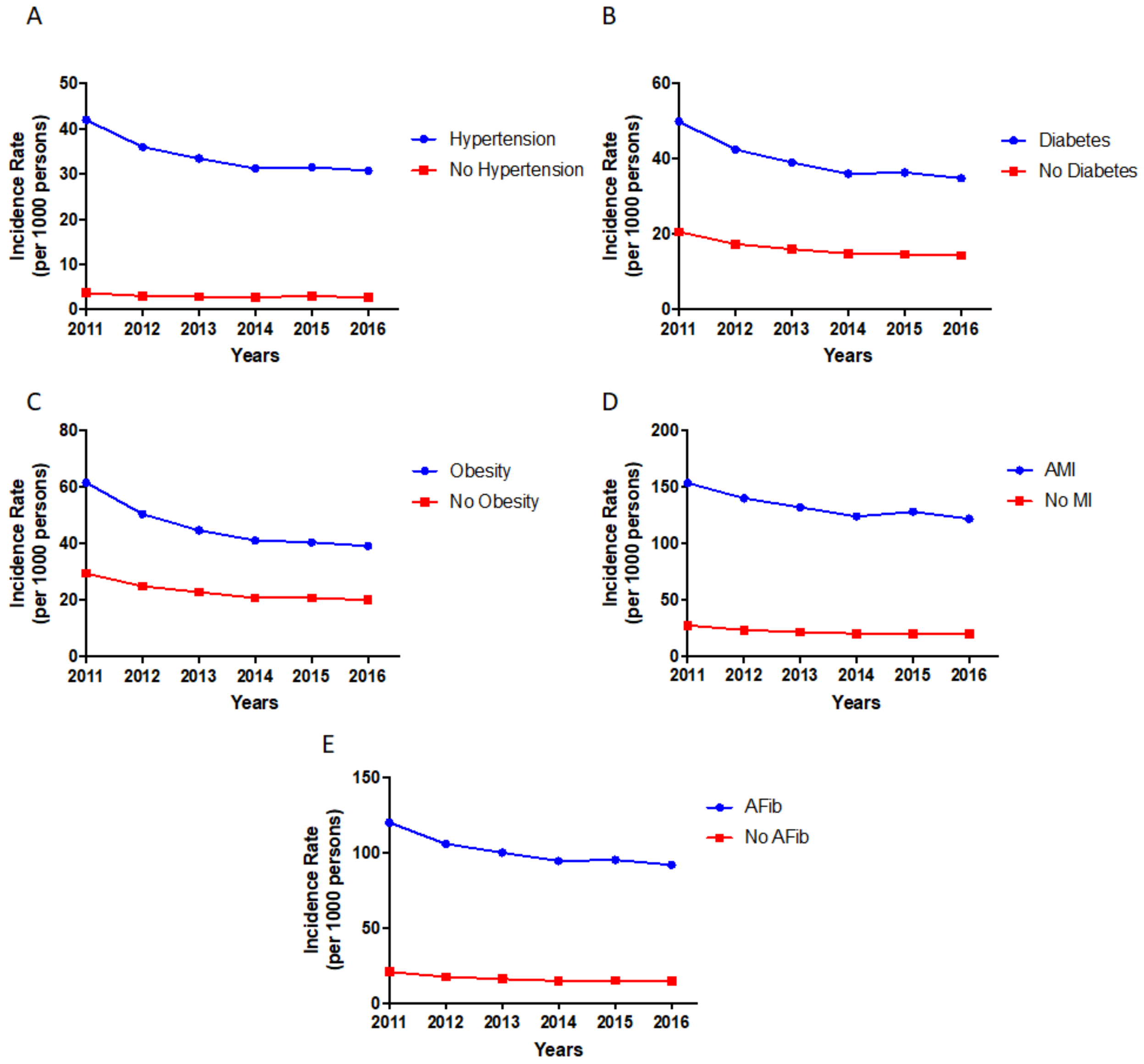

© 2020 Khera R et al. *JAMA Network Open*.

eFigure 4. Adjusted Incidence Rate Ratios for Heart Failure Among Beneficiaries With vs. Without Each Risk Factor Over Time, Hypertension (A), Diabetes (B), Obesity (C), Acute Myocardial Infarction (D), and Atrial Fibrillation (E), Yearly Trend. Poisson regression models were constructed to determine the association of each risk factor with HF incidence for each year with adjustment for age, sex, race, socioeconomic status, and all 5 risk factors. The p-value reflects trends in the incidence rate ratio over time. AF: Atrial fibrillation; AMI: Acute myocardial infarction. 2-year period of claims used to exclude prevalent heart failure.

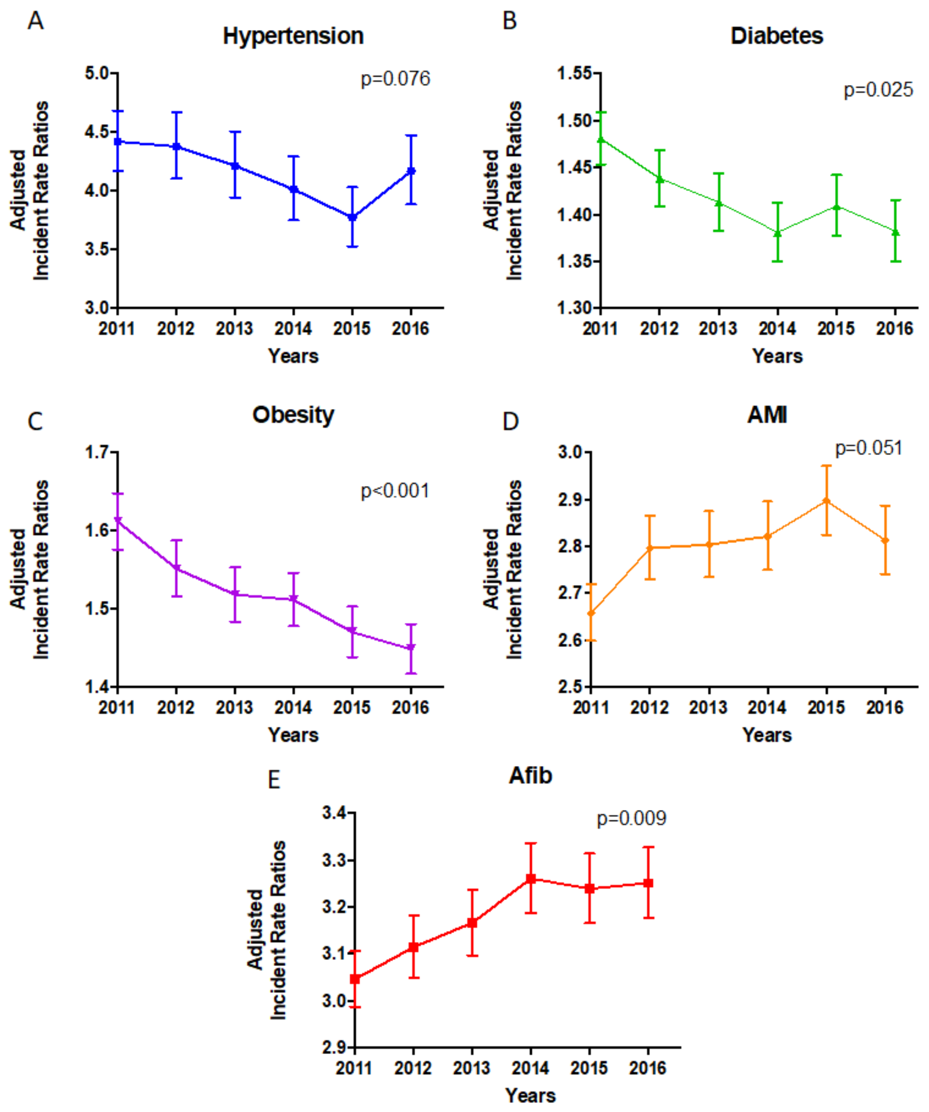

© 2020 Khera R et al. *JAMA Network Open*.

eReferences

1. Curtis LH, Whellan DJ, Hammill BG, et al. Incidence and prevalence of heart failure in elderly persons, 1994-2003. *Arch Intern Med.* 2008;168(4):418-424.
2. Jhund PS, Macintyre K, Simpson CR, et al. Long-term trends in first hospitalization for heart failure and subsequent survival between 1986 and 2003: a population study of 5.1 million people. *Circulation.* 2009;119(4):515-523.
3. Teng TH, Finn J, Hobbs M, Hung J. Heart failure: incidence, case fatality, and hospitalization rates in Western Australia between 1990 and 2005. *Circ Heart Fail.* 2010;3(2):236-243.
4. Ezekowitz JA, Kaul P, Bakal JA, Quan H, McAlister FA. Trends in heart failure care: has the incident diagnosis of heart failure shifted from the hospital to the emergency department and outpatient clinics? *Eur J Heart Fail.* 2011;13(2):142-147.
5. Zarrinkoub R, Wettermark B, Wandell P, et al. The epidemiology of heart failure, based on data for 2.1 million inhabitants in Sweden. *Eur J Heart Fail.* 2013;15(9):995-1002.
6. Gerber Y, Weston SA, Redfield MM, et al. A contemporary appraisal of the heart failure epidemic in Olmsted County, Minnesota, 2000 to 2010. *JAMA Intern Med.* 2015;175(6):996-1004.
7. Christiansen MN, Kober L, Weeke P, et al. Age-Specific Trends in Incidence, Mortality, and Comorbidities of Heart Failure in Denmark, 1995 to 2012. *Circulation.* 2017;135(13):1214-1223.
8. Khera R, Pandey A, Ayers CR, et al. Contemporary Epidemiology of Heart Failure in Fee-For-Service Medicare Beneficiaries Across Healthcare Settings. *Circ Heart Fail.* 2017;10(11).
9. Tsao CW, Lyass A, Enserro D, et al. Temporal Trends in the Incidence of and Mortality Associated With Heart Failure With Preserved and Reduced Ejection Fraction. *JACC Heart Fail.* 2018;6(8):678-685.
10. Conrad N, Judge A, Tran J, et al. Temporal trends and patterns in heart failure incidence: a population-based study of 4 million individuals. *Lancet.* 2018;391(10120):572-580.
11. Sulo G, Igland J, Overland S, et al. Heart failure in Norway, 2000-2014: analysing incident, total and readmission rates using data from the Cardiovascular Disease in Norway (CVDNOR) Project. *Eur J Heart Fail.* 2020;22(2):241-248.

© 2020 Khera R et al. *JAMA Network Open*.
